# Efficacy of acitretin in the treatment of reactive neutrophilic dermatoses in adult‐onset immunodeficiency due to interferon‐gamma autoantibody

**DOI:** 10.1111/1346-8138.15312

**Published:** 2020-03-23

**Authors:** Rujira Rujiwetpongstorn, Mati Chuamanochan, Napatra Tovanabutra, Romanee Chaiwarith, Siri Chiewchanvit

**Affiliations:** ^1^ Department of Internal Medicine Faculty of Medicine Chiang Mai University Chiang Mai Thailand; ^2^ Division of Infectious Diseases Department of Internal Medicine Faculty of Medicine Chiang Mai University Chiang Mai Thailand

**Keywords:** acitretin, adult‐onset immunodeficiency, interferon‐gamma autoantibody, reactive neutrophilic dermatoses, Sweet syndrome

## Abstract

Reactive neutrophilic dermatoses in adult‐onset immunodeficiency due to interferon‐γ autoantibody (AOID) are usually associated with concomitant active opportunistic infections. Data focusing on the treatment of these dermatoses with non‐immunosuppressive drugs are still lacking. The aim of this study was to assess the efficacy and safety of acitretin treatment of reactive neutrophilic dermatoses in AOID. We conducted a retrospective review of all patients with AOID who had reactive neutrophilic dermatoses and had been treated with acitretin from January 2008 to December 2018. In total, 23 patients had been diagnosed with AOID, with 27 episodes of reactive neutrophilic dermatoses (20 episodes of Sweet syndrome and seven episodes of generalized pustular eruption) and treated with acitretin. The median effective dose of acitretin was 10 mg/day. The mean initial response was 5.6 ± 2.3 days. The rash had almost or completely cleared within 2 weeks in 70.4% of patients. One case had developed a reversible acitretin‐induced liver injury with hepatocellular pattern. The median total duration of treatment was 3 months. In conclusion, this study demonstrates the potential role of acitretin as one of the treatments of choice for reactive neutrophilic dermatoses in AOID, attributable to its favorable response and good tolerability.

## Introduction

Adult‐onset immunodeficiency resulting from the acquired autoantibodies to interferon‐γ (IFN‐γ; AOID) is a well‐recognized disease. The IFN‐γ is an important cytokine in the cell‐mediated immune (CMI) response. Most reported cases have been from East Asia and South‐East Asia, including Thailand, Taiwan, China and Japan.^1^ Patients with AOID are susceptible to disseminated opportunistic infections (OI) from intracellular organisms, such as non‐tuberculous mycobacteria (NTM), *Talaromyces marneffei* and non‐typhoidal *Salmonella* spp.^1^, ^2^, ^3^ The most common organ involvement reported in patients with AOID is lymph node, followed by skin and soft tissue.^4^, ^5^, ^6^ Skin manifestations are categorized into skin infection and reactive dermatoses, particularly neutrophilic dermatoses, including Sweet syndrome, generalized pustular eruption and neutrophilic lobular panniculitis.^7^, ^8^ Most of the time, these neutrophilic reactive dermatoses occur concurrently with active OI, predominantly disseminated NTM infection.^8^

The drugs used in the treatment of these reactive neutrophilic dermatoses associated with AOID include systemic and/or topical corticosteroids, colchicine, dapsone, non‐steroidal anti‐inflammatory drugs, methotrexate and, rarely, acitretin.^7^, ^8^, ^9^ Acitretin, a second‐generation systemic retinoid, displays an inhibitory property of neutrophil migration,^10^, ^11^, ^12^ leading to several reports of its benefit in the treatment of neutrophilic dermatoses, particularly subcorneal pustular dermatosis, even in the absence of AOID.^13^, ^14^, ^15^ In conjunction with a lack of immunosuppressive effects, acitretin should be the preferred therapeutic option for patients with concomitant active OI. The benefit of acitretin in reactive neutrophilic dermatoses associated with NTM infection among four highly suspected cases with AOID has been previously reported from our institute.^9^ This study aims to demonstrate the efficacy and tolerability of acitretin in this clinical setting using a larger population size than previously reported.

## Methods

### Study subjects

A single‐institution, retrospective study of all patients with AOID at Maharaj Nakorn Chiang Mai Hospital, Thailand, from January 2008 to December 2018 was conducted. AOID had been diagnosed by: (i) a clinical background of disseminated OI that were supposed to be from a CMI defect; (ii) exclusion of other immunosuppressed states, such as HIV, malignancy or receiving immunosuppressive drugs; and (iii) demonstration of the antibodies to IFN‐γ, using enzyme‐linked immunosorbent assay.^3^

The reactive neutrophilic dermatoses had been confirmed by clinical manifestation combined with histopathological findings and negative microbiological study. Patients for whom acitretin was prescribed to treat reactive neutrophilic dermatoses were included in the study. This study was approved by the institutional review board of the Faculty of Medicine, Chiang Mai University (study code: MED‐2562‐06084).

### Data assessment

The medical records were reviewed and collected for demographic and baseline laboratory data, type of reactive neutrophilic dermatosis, distribution of skin lesions and associated symptoms. Details of treatment, including the initial and effective dose of acitretin, time to the initial response, duration of treatment until the rash almost or completely cleared, total duration of treatment, total follow‐up time, concomitant treatments and adverse events were assessed. The term “initial response” in this study was defined by a more than 30% attenuation of skin lesions compared with the baseline status before acitretin treatment.

### Statistical analyses

Data were analyzed and presented as numbers and percentages, means and standard deviations, or medians and interquartile ranges as appropriate.

## Results

A total of 128 cases with confirmed AOID were identified. Twenty‐eight patients had developed reactive neutrophilic dermatoses. Twenty‐three of them (nine males, 14 females) had these skin reactions treated with acitretin and were included in this cohort. The other five patients did not receive acitretin because of: (i) the presence of abnormal liver enzymes (one case); (ii) well‐controlled skin disease with previous treatments (i.e. colchicine and/or methotrexate) at other hospitals (three cases); and (iii) the patients refused to accept systemic treatment (one case). The mean age of patients at the time of diagnosis was 56 ± 7.6 years. Among them, four cases had had two episodes of reactive neutrophilic dermatosis contributing to a total of 27 episodes, which were finally included in this study. Of the 27 episodes, 20 (74.1%) were Sweet syndrome and seven (25.9%) were generalized pustular eruption. The most common areas of involvement were the lower extremities and acral areas (61.5% each). Fever had been reported in 15 of 23 episodes (65.2%). Baseline mean hemoglobin level was 10.1 ± 1.7 g/dL. Leukocytosis with neutrophilia had been observed in all patients. The mean level of inflammatory markers, namely erythrocyte sedimentation rate and C‐reactive protein, were 64.9 ± 26.3 mm/h and 101.5 ± 57.3 mg/L, respectively (Table 1). All of the reactive dermatosis episodes were associated with OI, especially NTM infection.

**Table 1:** Baseline characteristics of 23 patients

Feature	Value
Age, years (mean ± SD)	56 ± 7.6
Female, *n* (%)	14 (60.9)
Type of reactive neutrophilic dermatosis	
Sweet syndrome, *n* (%)	20 (74.1)
Generalized pustular reaction, *n* (%)	7 (25.9)
Distribution of skin lesions	
Lower extremities, *n* (%)	16 (61.5)
Acral area, *n* (%)	16 (61.5)
Upper extremities, *n* (%)	15 (57.7)
Trunk, *n* (%)	12 (46.2)
Head, *n* (%)	8 (30.8)
Neck, *n* (%)	5 (19.2)
Generalized, *n* (%)	3 (11.5)
Baseline laboratory profile (mean ± SD)	
Hemoglobin (g/dL)	10.1 ± 1.7
White blood cell (×10^3^ cells/µL)	19.7 ± 8.2
Neutrophils (%)	70.9 ± 11.2
Lymphocytes (%)	18.0 ± 8.9
Eosinophils (%)	5.7 ± 4.9
Platelets (×10^3^/µL)	422.0 ± 126.2
Creatinine (mg/dL)	1.0 ± 0.6
Aspartate aminotransferase (U/L)	21 ± 9.9
Alanine aminotransferase (U/L)	18.4 ± 10.4
Alkaline phosphatase (U/L)	160.8 ± 96.5
Erythrocyte sedimentation rate (mm/h)	64.9 ± 26.3
C‐reactive protein (mg/L)	101.5 ± 57.3

SD, standard deviation.

**Figure 1:**
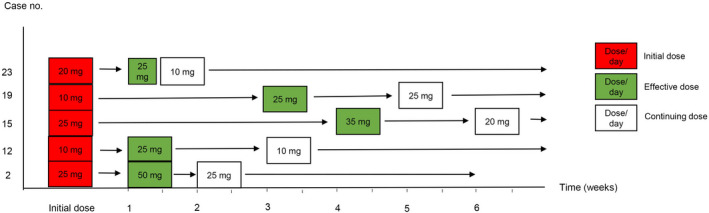
Timeline of five patients who required acitretin up‐dosing. The daily dose of acitretin is shown in the squares.

The initial dose of acitretin varied from 10 to 50 mg/day according to the severity and extent of the rash and the bodyweight of the patient, with a median daily dose of 10 mg (range, 10–25 mg; Table 2). A patient (case 3) who had been prescribed acitretin with an initial dose of 50 mg/day presented with extensive pustular eruption and progressed to exfoliative dermatitis resembling generalized pustular psoriasis. Twenty‐two of the 27 episodes (81.5%) had achieved satisfactory responses with the initial dose. Five episodes (18.5%) had needed up‐dosing, for which the starting dose ranged 10–25 mg/day and the time from initial dose to increased dose ranged 1–4 weeks (demonstrated as a timeline in Fig. 1).

**Table 2:** Acitretin treatment in 27 reactive neutrophilic dermatosis episodes in 23 patients

Feature	*n* (%)
Initially prescribed daily dose (mg)	
10	16 (59.3)
20	1 (3.7)
25	9 (33.3)
50	1 (3.7)
Effective daily dose (mg)	
10	14 (51.9)
25	10 (37.0)
35	1 (3.7)
50	2 (7.4)
Duration of treatment to clear/almost clear the disease with effective dose (week)	
≤1	4 (14.8)
≤2	19 (70.4)
≤3	23 (85.2)
≤4	26 (96.3)
≤5	27 (100)

The median effective dose was 10 mg/day (range, 10–25), mostly at low dose (Table 2). Taking bodyweight into account, the median effective daily dose by weight was 0.2 mg/kg per day (range, 0.2–0.5).

**Figure 2:**
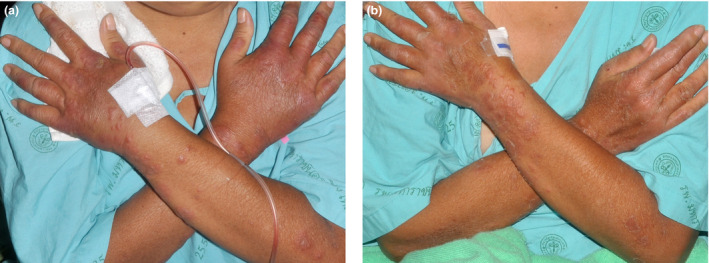
Initial response after acitretin treatment. (a) Sweet syndrome on dorsa of both hands and forearms in case 6 before acitretin treatment. (b) Initial response on day 3 after acitretin treatment in case 6.

**Figure 3:**
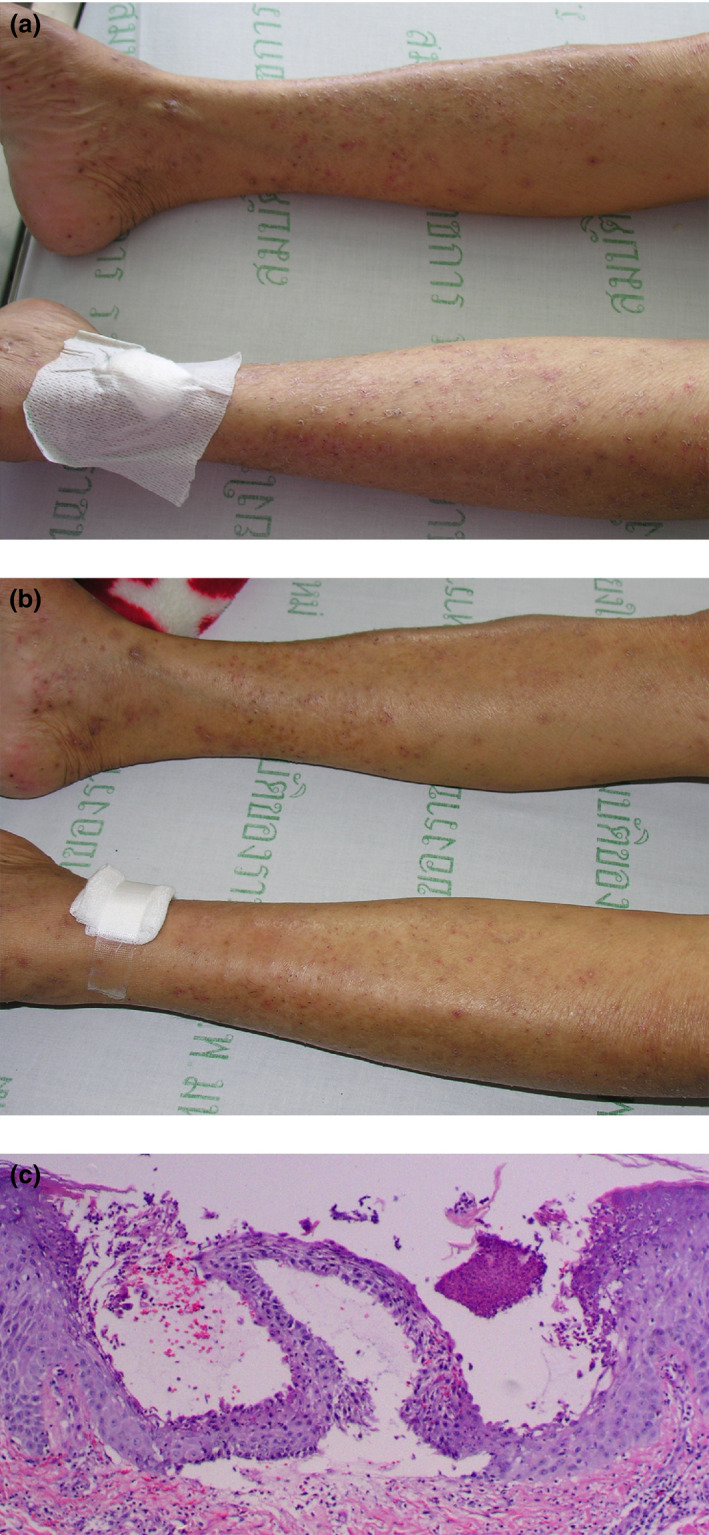
(a) Generalized pustular eruption on both legs in case 7. (b) Initial response on day 5 after acitretin treatment in case 7. (c) Histopathology shows subcorneal collection of neutrophils, spongiform pustules in the upper layer of the epidermis and infiltration of lymphocytes, neutrophils and rare eosinophils in the upper dermis (hematoxylin–eosin, original magnification ×100).

The mean time to the initial response of the effective dose, which was assessed only in eight inpatient cases, was 5.6 ± 2.3 days. The earliest response was noted within 3 days (Table 3, Figs 2,3). The rash had almost or completely cleared within the first follow‐up visit, ranging 1–5 weeks; most (70.4%) had cleared within a 2‐week period (Table 2). The median total duration of treatment was 3 months (range, 2–9), with a total follow‐up time of 14.5 months (range, 7–25).

**Table 3:** Details of clinical data and acitretin treatment in each patient

Case	Sex/age (years)	No. of episodes	Type of neutrophilic dermatosis	Pathogen of OI	Sites of OI	Effective dose of acitretin (mg/day)	Time to the initial response of the effective dose (only inpatient cases were assessed)	Duration of treatment to clear/almost clear the disease with the effective dose	Total duration of treatment (months)
1^†^	M/56	1	Sweet syndrome	*Mycobacterium avium* complex	LN, blood, skin and soft tissue	10	–	<10 days	9
2	F/49	1	Sweet syndrome	*Mycobacterium kansasii*	LN, blood	50^‡^	–	<1 week	2
3	M/54	1	Pustular reaction (exfoliative dermatitis)	NTM, not specified	LN, blood	50	–	<1 month	11
4	F/53	1	Sweet syndrome	NTM, not specified	LN, blood	10	7 days	12 days	1
5	F/50	1	Pustular reaction	NTM, not specified	LN	25	–	<1 week	12
		2	Pustular reaction	*Mycobacterium abscessus*	LN	25	–	<10 days	9
6	F/59	1	Pustular reaction	NTM, not specified	LN	10	–	<3 weeks	2
7	F/40	1	Sweet syndrome	Suspected NTM, not specified	LN	25	5 days	<1 month	4
8	F/61	1	Sweet syndrome	NTM, not specified	LN	10	–	<2 weeks	3
9	M/49	1	Sweet syndrome	Suspected NTM, not specified	LN	10	–	<1 week	6
10^†^	M/53	1	Sweet syndrome	NTM, not specified	LN	25	5 days	20 days	3
11^†^	M/64	1	Sweet syndrome	*Mycobacterium chelonae*	LN. blood, bone and joints	10	7 days	<18 days	2
12^†^	M/56	1	Sweet syndrome	NTM, not specified	LN	25^‡^	–	<2 weeks	2.5
13	M/58	1	Sweet syndrome	NTM, not specified	LN, blood	25	–	<2 weeks	9
		2	Sweet syndrome	NTM, not specified	LN	10	–	<20 days	2
14^†^	F/54	1	Sweet syndrome	*Mycobacterium gordonae*	LN	25	–	<2 weeks	6
		2	Sweet syndrome	NTM, not specified	LN	10	–	<2 weeks	11
15	F/52	1	Sweet syndrome	NTM, not specified	LN	35^‡^	–	<2 weeks	3
16	F/60	1	Sweet syndrome	Suspected *M. tuberculosis*	LN, spleen	10	–	<2 weeks	1
17	F/44	1	Pustular reaction	*M. abscessus*,* M. chelonae *complex and *M. tuberculosis*	LN, spleen	10	3 days	1 week	15
18	F/53	1	Pustular reaction	NTM, not specified	LN	25	–	<2 weeks	11
19^†^	F/75	1	Sweet syndrome	NTM, not specified	LN, skin and soft tissue	10	–	<2 weeks	1.5
		2	Sweet syndrome	NTM, not specified	LN	25^‡^	–	<2 weeks	6
20	F/59	1	Pustular reaction	Suspected NTM, not specified	LN	10	5 days	2 weeks	1
21^†^	F/52	1	Sweet syndrome	Suspected NTM, not specified	LN	10	–	<2 weeks	1.5
22	M/60	1	Sweet syndrome	NTM, not specified	LN	10	10 days	<5 weeks	2
23	M/53	1	Sweet syndrome	*M. chelonae*	LN	25^‡^	3 days	<1 month	10

^†^ The skin lesions improved before antimicrobial therapy. ^‡^Acitretin was up‐dosed from initial dose in order to achieve the clinical response. LN, lymph node; NTM, non‐tuberculous mycobacteria; OI, opportunistic infection.

As previously described, all skin episodes were associated with active OI that required antimicrobial therapy. Of the 27 episodes, 17 (63%) were treated with antimicrobial therapy concurrently with acitretin, whereas 10 (37%) were treated with antimicrobial therapy after acitretin with a time lag due to the laboratory methods for causal pathogen identification. In the latter group, the reactive neutrophilic dermatosis had completely resolved before antimicrobial initiation.

Six patients had relapse of neutrophilic dermatoses after acitretin was stopped. The relapse in two cases (cases 9 and 12) resulted from non‐adherence to acitretin treatment while continuing the same concomitant antimicrobial agents. The lesions resolved promptly after re‐administration of acitretin. Four other patients (cases 5, 13, 14 and 19) had relapse of neutrophilic dermatoses after completion of the first episode of the treatment; all relapses were associated with new episodes of NTM infection.

There were three cases of reactive neutrophilic dermatosis treated with other anti‐inflammatory or immunosuppressive agents before initiation of acitretin. The first case (case 5) had received 40 mg/day prednisolone and 100 mg/day dapsone with only moderate improvement. Acitretin 25 mg/day had been added, with an impressive response within 1 week. The second one (case 8) had achieved only a partial response to a 2‐week period of colchicine administration of 1.2 mg/day. Ten milligrams per day of acitretin had then been prescribed, with an obvious response in 2 weeks. The last patient (case 23) had failed to respond to prednisolone at a dose of 20 mg/day, and a notable improvement had been observed within 3 days after acitretin administration of 25 mg/day.

Acitretin was well tolerated, except in one patient (case 7), who had developed a reversible acitretin‐induced liver injury with hepatocellular pattern (a 10‐fold rise above the upper normal limit of alanine transaminase) at the 6th month of administration of 10 mg/day acitretin. Acitretin had then been discontinued and the liver enzyme had returned to normal within 1 month.

## Discussion

This study shows the considerable efficacy of acitretin in the treatment of neutrophilic dermatoses associated with AOID. The rash had almost or completely cleared within 2 weeks with 10–25 mg/day acitretin in most of the patients. The mean time to initial response was 5.6 days, which is comparable with the 7–10 days reported for pustular psoriasis treatment.^11^ None of the cases in this cohort failed to respond to acitretin treatment or required additional topical and/or systemic drugs to control the skin reaction.

One‐fifth of our cases had failed to respond to the initial dose. Interestingly, only Sweet syndrome had been reported as the skin reaction in this group, implying that Sweet syndrome may be more difficult to treat with acitretin than pustular eruption. As might be expected, all patients had responded well to up‐dosing, which is concordant with previous data in psoriasis treatment, showing that the treatment response was dose‐dependent.^11^

Data from our cohort show that there were seven cases whose skin reaction had definitely resolved with acitretin treatment before initiation of antimicrobial therapy. Moreover, in two cases whose neutrophilic dermatoses had flared due to non‐adherence to acitretin treatment, the rash had improved after acitretin had been restarted, in spite of continuing the same antimicrobial agents. A number of patients with a poor response to other anti‐inflammatory drugs, for example, prednisolone, colchicine and dapsone, had shown a rapid response to acitretin.

To date, the pathophysiology of reactive neutrophilic dermatoses in patients with AOID remains to be elucidated, including the response to OI, an overactivation of innate immunity to compensate for any defect in the CMI pathway and the dysregulation of cytokine pathways involving T‐helper (Th)1 cytokines or the interleukin‐23/Th17 axis.^8^, ^16^, ^17^ These proposed hypotheses may result in aggregation and accumulation of neutrophils in the skin. Acitretin has an inhibitory effect on neutrophil migration, supporting its potential role in the treatment of this reactive neutrophilic dermatosis. The duration of the treatment should depend on the infectious control.^11^, ^18^

Concerning the safety profile of acitretin, only one patient in our cohort (3.7%) had suffered acitretin‐induced hepatotoxicity that was completely reversed after discontinuation of acitretin. This incidence is considerably lower than the reported prevalence, which ranged 21.3–30.5%.^19^, ^20^ Other adverse reactions, such as lipid abnormalities, cheilitis and dry skin, were not reported in our cohort. The serious concern of acitretin prescription, namely a teratogenic effect, is less worrisome because patients in this setting are generally not at a child‐bearing age.

The following drugs have been used in the treatment of neutrophilic/pustular dermatoses in AOID, including corticosteroid, methotrexate, potassium iodide, colchicine and dapsone.^18^, ^21^ The first three drugs have an immunosuppressive effect that is not good for the associated disseminated OI conditions. Furthermore, the most common associated OI is NTM infection, in which a macrolide is frequently used. Because macrolide and colchicine are metabolized through CYP3A4, co‐administration of these two agents can cause fatal toxicity, such as pancytopenia, rhabdomyolysis or even multiorgan failure.^22^, ^23^ Lastly, dapsone is also not a preferred option because the patients are usually anemic.

There were some limitations to our study. First, this was a retrospective, single‐institutional and uncontrolled study, due to the rarity of the studied condition. Second, determination of the precise time to the clinical response was possible only in inpatient cases. Moreover, the majority of our patients were ambulatory cases; the reported response time might have been underestimated. Further multicenter, randomized controlled studies with regular and short follow‐up periods should be conducted to assess the reproducibility of the results found in this study.

In summary, acitretin is suggested as the first‐line treatment in reactive neutrophilic dermatoses associated with AOID, due to its efficacy, tolerability and absence of immunosuppressive effect, as evidenced by our 10 years of experience. However, additional randomized controlled studies are crucial to confirm this conclusion.

## Conflict of Interest

None declared.
